# Immunopathology of galectin-3: an increasingly promising target in COVID-19

**DOI:** 10.12688/f1000research.25979.2

**Published:** 2020-09-28

**Authors:** John L. Caniglia, Swapna Asuthkar, Andrew J. Tsung, Maheedhara R. Guda, Kiran K. Velpula

**Affiliations:** 1 Departments of Cancer Biology and Pharmacology, University of Illinois College of Medicine at Peoria, Peoria, IL, USA; 2 Department of Neurosurgery, University of Illinois College of Medicine at Peoria, Peoria, IL, USA; 3 Illinois Neurological Institute, Peoria, IL, USA; 4 Department of Pediatrics, University of Illinois College of Medicine at Peoria, Peoria, IL, USA

**Keywords:** COVID-19, galectin, cytokines; ARDS, fibrosis, sialic acid, galectin-3

## Abstract

The pandemic brought on by the outbreak of severe acute respiratory syndrome coronavirus 2 (SARS-CoV2) has become a global health crisis, with over 22 million confirmed cases and 777,000 fatalities due to coronavirus disease 2019 (COVID-19) reported worldwide. The major cause of fatality in infected patients, now referred to as the “Cytokine Storm Syndrome” (CSS), is a direct result of aberrant immune activation following SARS-CoV2 infection and results in excess release of inflammatory cytokines, such as interleukin (IL)-1, tumor necrosis factor α (TNF-α), and IL-6, by macrophages, monocytes, and dendritic cells. Single cell analysis has also shown significantly elevated levels of galectin 3 (Gal-3) in macrophages, monocytes, and dendritic cells in patients with severe COVID-19 as compared to mild disease. Inhibition of Gal-3 reduces the release of IL-1, IL-6, and TNF-α from macrophages *in vitro*, and as such may hold promise in reducing the incidence of CSS. In addition, Gal-3 inhibition shows promise in reducing transforming growth factor ß (TGF-ß) mediated pulmonary fibrosis, likely to be a major consequence in survivors of severe COVID-19. Finally, a key domain in the spike protein of SARS-CoV2 has been shown to bind *N-*acetylneuraminic acid (Neu5Ac), a process that may be essential to cell entry by the virus. This Neu5Ac-binding domain shares striking morphological, sequence, and functional similarities with human Gal-3. Here we provide an updated review of the literature linking Gal-3 to COVID-19 pathogenesis. Dually targeting galectins and the Neu5Ac-binding domain of SARS-CoV2 shows tentative promise in several stages of the disease: preventing viral entry, modulating the host immune response, and reducing the post-infectious incidence of pulmonary fibrosis.

## Introduction

Galectin 3 (Gal-3) is a carbohydrate-binding protein that exhibits pleiotropic effects throughout the body, including the modulation of apoptosis, cell migration and adhesion, angiogenesis, tumorigenesis, and post-injury remodeling ( Chen & Kuo, 2016; Elola *et al.,* 2018; Nangia-Makker *et al.*, 2018). It is most highly expressed in myeloid cells (macrophages, dendritic cells, neutrophils, and monocytes), as well as epithelial cells, endothelial cells, and fibroblasts ( Diaz-Alvarez & Ortega, 2017). When secreted by myeloid cells, Gal-3 and other galectins can act as modulators of cytokine expression by immune cells, and also as orchestrators of the damage associated molecular pattern (DAMP) system ( Sato *et al.,* 2009). Recent discoveries specific to viral infections have begun to shed light on its role as well ( Wang *et al.,* 2019). For example, in HIV and HTLV, Gal-3 serves as an attachment factor that facilitates viral entry into T-cells ( Wang *et al.,* 2019). HIV infection also induces further Gal-3 expression through activation of NF-kB dependent pathways ( Okamato *et al.,* 2019). Secreted Gal-3 then mediates a number of deleterious effects. In particular, Gal-3 has been shown during infection to induce a dysregulated pattern expression of pro-inflammatory cytokine expression via the JAK/STAT1, ERK, and AKT signaling pathways ( Nita-Lazar *et al.,* 2015). The cytokine profile observed includes tumor necrosis factor α (TNFα), interleukin (IL)-1β, and IL-6, among others ( Nita-Lazar *et al.,* 2015). Gal-3 is also a known agonist of toll like receptor 4 (TLR4) and nuclear factor kappa beta (NF-kB) dependent pathways, which are well characterized and potent inducers of inflammation during infection ( Yip *et al.,* 2017; Zhou *et al.,* 2018). Patients suffering from severe coronavirus disease 2019 (COVID-19) show highly elevated levels of Gal-3, TNFα, IL-1β, and IL-6, as compared to those with moderate disease ( De Biasi *et al.,* 2020; Wang *et al.,* 2020a). Inhibition of Gal-3 significantly reduces the levels of these cytokines, and so may show promise in reducing inflammatory sequelae associated with COVID-19 ( De Biasi *et al.,* 2020; Kalfaoglu *et al.,* 2020; Liu *et al.,* 2020).

The continued lack of an effective standard of care for treating patients with COVID-19 has brought on an urgent need to identify effective therapies. In a prior review article, we had discussed promising indications for Gal-3 targeted therapy in the treatment of COVID-19, with the goal of inspiring further research on the topic ( Caniglia *et al.,* 2020). In recent months, however, a substantial amount of new evidence has emerged that further links Gal-3 to severe COVID-19 infection. As such, the authors see a need to achieve two aims in this review: highlighting novel discoveries to expand upon previously discussed treatment indications, and to detail a further potential role for anti-galectin therapy in reducing post-infectious pulmonary fibrosis. This article may be particularly useful for immunologists studying COVID-19, as well as any researchers with a structural or functional focus on galectins.

## SARS-CoV2: host cell attachment and entry

**Figure 1.**
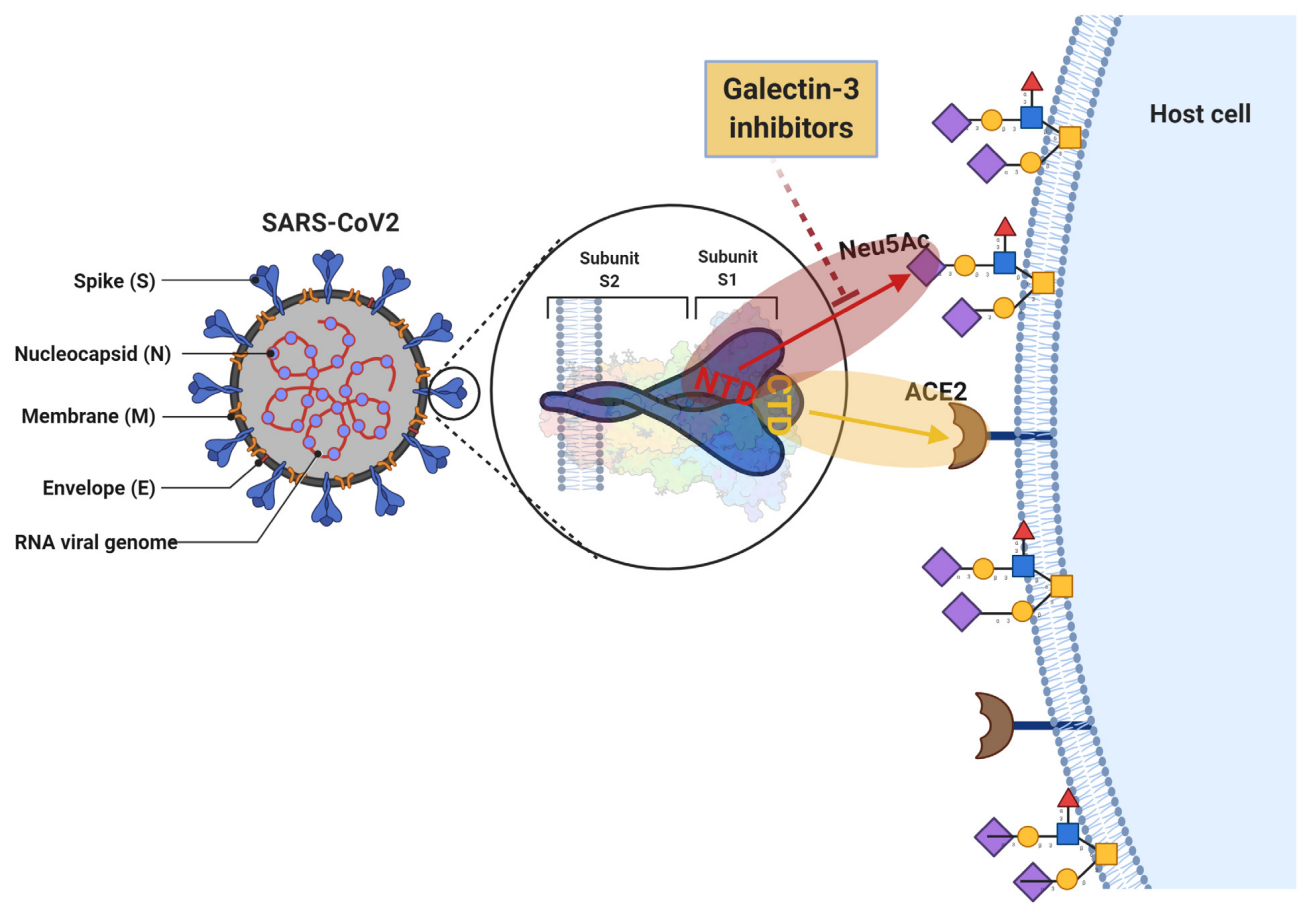
A dual attachment model for SARS-CoV2. Evidence has shown that a pocket in the NTD of SARS-CoV2 is capable of binding *N*-acetylneuraminic acid (Neu5Ac). This strongly supports a dual attachment model for SARS-CoV2, where NTD-Neu5Ac interactions facilitate initial host cell recognition by the virus and stabilize its entry via ACE2 receptors.

A critical step prior to viral infection is the entry of the virus into host cells, a process that in severe acute respiratory syndrome coronavirus 2 (SARS-CoV2) is mediated by the S1 subunit of the spike protein ( Blaas, 2016; Zhai *et al.,* 2020). Within coronaviridae, it is commonplace to refer to the S1 protein as consisting of two distinct regions: the C-terminal domain (CTD) and N-terminal domain (NTD) ( Li, 2016). In most cases, the CTD binds peptide receptors and the NTD binds sugar receptors ( Li, 2016). The main entry mechanism of SARS-CoV2 has been shown to be via the CTD binding to angiotensin converting enzyme receptor 2 (ACE2) receptors ( Wang *et al.,* 2020b). Until recently, the role of the NTD has been largely overlooked. A study from Baker *et al.* has shown evidence that SARS-CoV2 also binds N-acetylneuraminic acid (Neu5Ac), with this interaction being mediated by the NTD of the S1 subunit ( Baker *et al.,* 2020). This is the first *in vitro* evidence of this occurring, although several prior bioinformatics and modeling studies have hypothesized that a Neu5Ac binding site exists, with one suggesting its affinity for Neu5Ac (0.88) is only slightly lower than that of influenza hemagglutinin (0.94) ( Alban *et al.,* 2020; Behloul *et al.,* 2020; Fantini *et al.,* 2020; Kim, 2020; Milanetti *et al.,* 2020; Robson, 2020). Binding of sialic acids by the NTD is the main entry mechanism in several other coronaviruses known to infect humans, most notably members of the bovine coronavirus family ( Li, 2015). Additionally, the closely related middle eastern respiratory syndrome coronavirus (MERS-CoV) has been shown to exhibit a dual attachment model similar to SARS-CoV2, where the CTD binds a peptide receptor and the NTD binds sialic acids ( Li *et al.,* 2017). Depletion of sialic acids with neuraminidase inhibitors prevented MERS-CoV infection of Calu-3 human airway cells, indicating that NTD-targeted therapies may be effective in preventing cell entry by coronaviruses possessing this function ( Li *et al.,* 2017). Additionally, a neutralizing antibody against the SARS-CoV2 S1-NTD has been shown to completely inhibit cell entry by the virus ( Chi *et al*., 2020). This indicates the NTD region is essential for viral entry and a promising therapeutic target ( Chi *et al*., 2020). The dual mechanism by which SARS-CoV2 may enter host cells is seen in Figure 1.

The binding of Neu5Ac may also explain the greater infectivity of SARS-CoV2 as compared to SARS-CoV ( Alban *et al.,* 2020). While the CTD of SARS-CoV2 has been shown to exhibit higher affinity for ACE2 receptors than that of SARS-CoV, this is likely insufficient to fully explain the marked disparity in transmissibility ( Tai *et al.,* 2020). The NTD of SARS-CoV2 has been rigorously analyzed and compared to both human galectins and the NTD of other coronaviruses ( Behloul *et al.,* 2020). Behloul *et al.* found that while SARS-CoV2 and SARS-CoV share 74.75% similarity in the CTD, they exhibit just 52.69% similarity in the NTD region ( Behloul *et al.,* 2020). This is particularly noteworthy when viewed together with the findings that despite SARS-CoV2 being able to bind Neu5Ac *in vitro*, the same domain on SARS-CoV did not exhibit this ability ( Baker *et al.,* 2020). Modeling studies comparing the NTD of SARS-CoV2 and SARS-CoV have led to the same conclusion ( Behloul *et al.,* 2020). The far greater abundance of Neu5Ac in the human body as compared to ACE2 receptors, particularly at common viral entry points such as the nasopharynx and oral mucosa, may explain the high transmissibility of SARS-CoV2 ( Barnard *et al.,* 2019).

Several studies to date have referred to the “galectin fold” present on the NTD of coronaviruses ( Behloul *et al.,* 2020; Li, 2016; Li *et al.,* 2017; Peng *et al.,* 2011; Peng *et al.,* 2012; Tortorici *et al.,* 2019). The structures of Gal-3 and the S1-NTD of betacoronaviridae are so similar, in fact, that it is hypothesized that coronaviruses incorporated a host galectin gene into their genome (and then the NTD) at some point in their evolution ( Caniglia *et al.,* 2020; Li, 2015). Structural analysis comparing the SARS-CoV2 NTD to Gal-3 resulted in a Z-score of 6 (p < 0.00001), indicating a high degree of similarity between the structures ( Behloul *et al.,* 2020). In fact, human Gal-3 was shown to be equally similar to SARS-CoV2 NTD as the NTD of NL63-CoV and infectious bronchitis coronavirus, accounting for both sequence and structure ( Behloul *et al.,* 2020). Given the high degree of structural and promising sequence similarity (12%) of the NTD with Gal-3, it may be possible that existing Gal-3 inhibitors possess dual-binding capabilities ( Behloul *et al.,* 2020). Such a mechanism shows promise in reducing viral entry to host cells ( Milanetti *et al.,* 2020).

## Gal-3 in severe infection: promoting immunologic sequelae of COVID-19

The major cause of death in patients infected with SARS-CoV and MERS-CoV infection was found to be the “Cytokine Storm Syndrome” (CSS), and this is likely to be the case in COVID-19 as well ( Channappanavar & Perlman, 2017; Zhang *et al.,* 2020). CSS develops due to hyper-activation of macrophages, monocytes, and dendritic cells, which are stimulated to release a variety of inflammatory mediators including IL-1, IL-6, and TNF-α ( Zhang *et al.,* 2020). This in turn leads to systemic organ dysfunction that may result in death ( England *et al.,* 2020). Notably, a study of nearly 4,000 patients has found the levels of IL-1, IL-6, and TNF-α to be significantly elevated in the sera of patients suffering from severe COVID-19 as compared to those with mild disease ( Wang *et al.,* 2020a). Similar findings were reported in a cohort of over 1,5000 patients, where serum IL-6 and TNF-α were found to be independent predictors of disease severity and mortality in COVID-19 ( Del Valle *et al.,* 2020). This data speaks to the urgency of identifying therapeutics to reduce the incidence of CSS ( Del Valle *et al.,* 2020; Wang *et al.,* 2020a).

**Figure 2.**
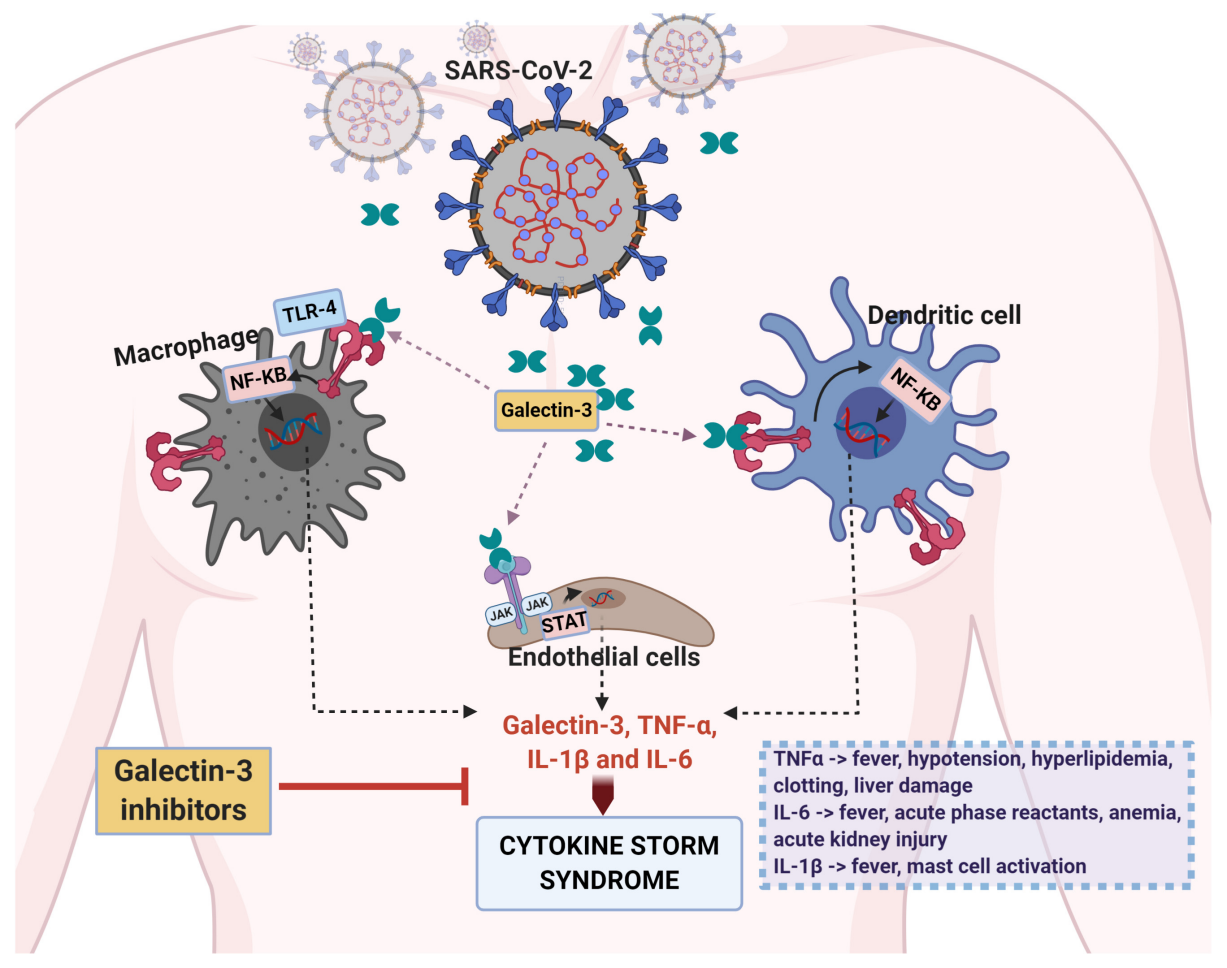
Gal-3 may amplify the cytokine storm syndrome associated with severe COVID-19. During severe SARS-CoV2 infection, increased plasma concentrations of Gal-3 are observed in circulating macrophages, monocytes, and dendritic cells. When secreted, Gal-3 can then agonize TLR4 receptors on their surfaces and induce the release of inflammatory cytokines such as IL-1, IL-6, and TNF-α. This process also results in the secretion of further Gal-3, resulting in a positive feedback loop that may contribute to the development of CSS.

There is a plethora of evidence that makes Gal-3 a promising target to achieve this aim. First, the most concerning sequelae of CSS is evolution to acute respiratory distress syndrome (ARDS), a condition which often leads to respiratory failure despite proactive measures such as mechanical ventilation and intubation ( Vabret *et al.,* 2020). Elevated serum levels of Gal-3 are significantly associated with worse outcomes and lower survival in patients suffering from ARDS ( Xu *et al.,* 2017). Additionally, significantly elevated levels of Gal-3 have been shown in the serum of patients suffering from severe COVID-19 as compared to those with mild disease ( De Biasi *et al.,* 2020). On a cellular level, Gal-3 was shown to be most elevated in immune cells during severe COVID-19 ( Kalfaoglu *et al.,* 2020) The highest levels of Gal-3 were seen in infected macrophages, monocytes, and dendritic cells, the very cells responsible for initiating CSS ( Liu *et al.,* 2020). A pathway through which Gal-3 may contribute to the development of CSS is detailed in Figure 2.

Several studies to date have shown the effects of anti-Gal-3 therapy on cytokine release ( Chen *et al.,* 2015; Chen *et al.,* 2018; Ren *et al.,* 2019; Yip *et al.,* 2017). Significant reductions in IL-1, IL-6, and TNF-α secretion by dendritic cells has been observed upon silencing of Gal-3 ( Chen *et al.,* 2015). In models of traumatic brain injury and spinal cord injury, treatment with anti-Gal-3 antibodies and the Gal-3 inhibitor GB1107, respectively, both led to significant reductions in the systemic levels of IL-1, IL-6, and TNF-α ( Ren *et al.,* 2019; Yip *et al.,* 2017). Gal-3 K/O has also been shown to decrease both NF-kB activation and HIV viral replication in infected cells ( Okamato *et al.,* 2019). Lastly, in mice infected with H5N1 influenza virus, Gal-3 K/O led to a significant reduction of IL-1ß secretion by macrophages and improved survival rate as compared to controls ( Chen *et al.,* 2018). These findings are due to Gal-3’s known role as an alarmin of the innate immune system, triggering the release of inflammatory cytokine, such as TNF-α and IL-6 from monocyte-derived cells during infection or other inflammatory insults ( Mishra *et al.,* 2013; Yip *et al.,* 2017). The enhanced secretion of cytokines likely occurs through TLR4/NF-kB mediated pathways ( Yip *et al.,* 2017; Zhou *et al.,* 2018). With all this information taken together, Gal-3 inhibition shows promise in reducing the incidence and symptoms of CSS.

## Gal-3 post-infection: pathologic fibrosis

It is well known that persistent viral infections are a risk factor for the subsequent development of pulmonary fibrosis ( Sheng *et al.,* 2020). A study found that tests for SARS-CoV2 RNA in the serum of infected individuals did not become negative until a median of 24 days post-symptom onset, with some individuals remaining positive even greater than a month from the beginning of symptoms ( Gombar *et al.,* 2020). Additionally, in a cohort of recovered COVID-19 patients in Italy, 87.4% reported persistent symptoms, most notably fatigue and dyspnea, at an average of 60.3 days post-infection ( Carfi *et al.*, 2020). This indicates that for some, persistent post-viral inflammation may result in deleterious changes such as pulmonary fibrosis ( Crisan-Dabija *et al.*, 2020). Findings such as these have led to the question of whether or not anti-fibrotic therapy would be beneficial for such patients ( George *et al.*, 2020).

In SARS-CoV infection, particularly in patients who suffered from ARDS, marked pulmonary fibrosis was found in a cohort of patients following prolonged infection ( Ye *et al.,* 2007). Though long term outcomes remain to be seen, lung tissue in the acute phase of COVID-19 shows similar changes ( Xu *et al.,* 2020a). Following a 24 hour incubation of SARS-CoV2, human airway cells showed upregulation of ACE2, vascular endothelial growth factor (VEGF), connective tissue growth factor (CTGF), fibronectin (FN), and transforming growth factor ß (TGF-ß), a molecular signature highly similar to that of patients with diagnosed pulmonary fibrosis ( Xu *et al.,* 2020a). It is believed that a large number of COVID-19 patients will go on to develop pulmonary fibrosis, and that these changes are mediated by a number of cytokines including TGF- ß, IL-1, IL-6, and TNF-α ( Delpino & Quarleri, 2020).

**Figure 3.**
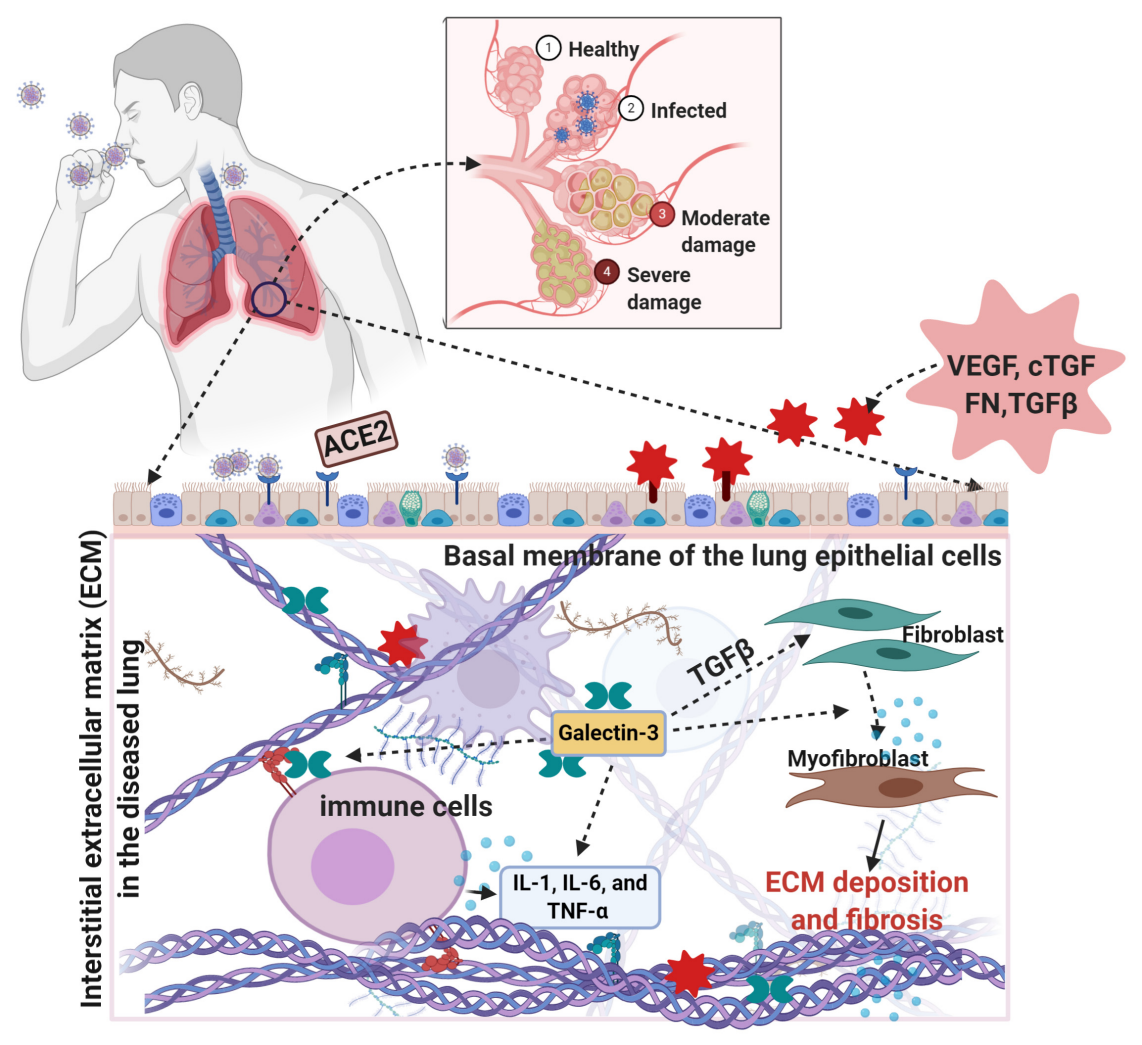
Gal-3 contributes to a pro-fibrotic microenvironment in COVID-19. During SARS-CoV2 infection, transcriptional upregulation of VEGF, TGF-ß, and fibronectin (FN) is seen in the pulmonary epithelium, creating a pro-fibrotic microenvironment. Secretion of Gal-3 by macrophages contributes to fibrosis by increasing the expression of TGF-ß receptors on the surface of fibroblasts. The fibroblasts and myofibroblasts are then activated by TGF-ß mediated signaling, stimulating the deposition of extracellular matrix and collagen that leads to fibrotic damage. Cytokines induced by Gal-3 expression such as IL-1, IL-6, and TNF-α further accelerate this process.

The role of Gal-3 as a mediator of lung fibrosis has long been studied since the discovery that its levels are elevated in alveolar macrophages following lung injury ( Kasper & Hughes, 1996; Nishi *et al.,* 2007). Higher levels of Gal-3 have now been extensively associated with the development of restrictive lung diseases ( Ho *et al.,* 2016). Following cellular stress, the secretion of Gal-3 by macrophages upregulates TGF-ß receptors on fibroblasts and myofibroblasts ( Henderson *et al.,* 2008). This in turn activates these cells, initiating the formation of granulation tissue (via collagen deposition) that is eventually remodeled to a fibrous scar ( Henderson *et al.,* 2008; Mackinnon *et al.,* 2012). This Gal-3 mediated pathway is widespread throughout the body and fundamental to the development of fibrotic change in the liver, kidneys, and heart as well ( Hara *et al.,* 2020). Gal-3 mediated fibrosis often has deleterious effects; for example, pathologic scar formation is the likely explanation for serum Gal-3’s utility as an independent predictor of mortality and heart failure post-myocardial infarction ( Asleh *et al.,* 2019). The mechanism by which Gal-3 may contribute to post-infectious pulmonary fibrosis in COVID-19 patients can be seen in Figure 3.

Gal-3 inhibitors show promise in limiting fibrotic change following lung injury. In a model of adenovirus induced lung injury, Gal-3 K/O mice showed significant reductions in lung fibrosis and ß-catenin activation, indicating the beneficial effects were mediated via interruption of TGF-ß signaling ( Mackinnon *et al.,* 2012). Treatment with the drug TD139 showed significant reductions in these parameters as well following bleomycin-induced pulmonary fibrosis ( Mackinnon *et al.,* 2012). This drug (now referred to as GB0139) was well tolerated in phase I/IIa trials in the treatment of idiopathic pulmonary fibrosis (IPF) and is now in phase IIb trials ( Saito *et al.,* 2019). An additional indication for this drug may be in reducing the post-viral development of pulmonary fibrosis ( Mackinnon *et al.,* 2012). The drug TD139 has recently begun phase II trials for the treatment of COVID-19, the first clinical trial of a galectin inhibitor in COVID-19 to date ( University of Edinburgh DEFINE trial, 2020).

## Conclusions and future directions

In summary, Gal-3 is a lectin that exhibits a pleiotropic role in mediating the acute and chronic consequences of infection and inflammation. Multiple studies have shown Gal-3 to be highly upregulated in patients suffering from severe COVID-19 ( De Biasi *et al.,* 2020; Kalfaoglu *et al.,* 2020; Liu *et al.,* 2020). On a cellular level, Gal-3 is most highly expressed in monocytes, macrophages, and dendritic cells during severe COVID-19 infection ( Liu *et al.,* 2020). CSS complicated by the development of ARDS is the major cause of fatality in COVID-19 patients ( Xu *et al.,* 2020b; Zhai *et al.,* 2020; Zhang *et al.,* 2020). This process is chiefly mediated by the release of IL-1, IL-6, and TNF-α from macrophages, monocytes, and dendritic cells ( Zhang *et al.,* 2020). Gal-3 inhibition has been shown to reduce the release of these cytokines from immune cells ( Chen *et al.,* 2015; Ren *et al.,* 2019; Yip *et al.,* 2017). Additionally, high Gal-3 is directly associated with worse outcomes and lower survival in ARDS patients ( Xu *et al.,* 2017).

A key domain in the spike protein exhibits a high degree of morphological and sequence similarity to human Gal-3 ( Behloul *et al.,* 2020). This NTD has been shown to bind Neu5Ac *in vitro*, an interaction that likely explains the high infectivity of SARS-CoV2 and may be essential for cell entry ( Alban *et al.,* 2020; Barnard *et al.,* 2019; Baker *et al*., 2020). Inhibitors of Gal-3 that target regions of structural overlap with the NTD may possess dual binding capabilities, exhibiting a novel mechanism by which to inhibit viral entry ( Milanetti *et al.,* 2020).

Lastly, pulmonary fibrosis has been observed following SARS-CoV infection and is likely to be a major complication in survivors of COVID-19 that is cytokine-mediated ( Delpino & Quarleri, 2020; Xu *et al.,* 2020b; Ye *et al.,* 2007). Among other mediators, elevated levels of TGF-ß have been observed following SARS-CoV2 infection ( Xu *et al.,* 2020a). Gal-3 secreted by macrophages during injury promotes the upregulation of TGF-ß receptors, leading to fibroblast activation and collagen deposition ( Delpino & Quarleri, 2020). Gal-3 inhibition has been shown to reduce adenovirus-induced lung fibrosis, and an inhibitor is currently in Phase IIb clinical trials for IPF treatment ( Mackinnon *et al.,* 2012; Saito *et al.,* 2019). The indications for targeting Gal-3 in the treatment of COVID-19 are widespread. Processes directly mediated or affected by Gal-3 have been shown to be deleterious in several stages of the disease process. As such, Gal-3 represents a highly promising target for COVID-19 treatment that should urgently be investigated.

## Literature search methodology

### Eligibility criteria

This review consists of original studies that provided information about SARS-CoV2, Gal-3, or Gal-3 inhibitors. Compiled results from both *in vivo*, *in vitro,* and clinical studies were used for analysis. Studies with only an abstract or no full-text available were excluded from the review.

### Search methodology

To retrieve primary literature, electronic searches were performed on PubMed and Google Scholar. A list of search terms can be seen in Table 1.

**Table 1.** Search strategy for our literature review.

Database	Search Queries
**PubMed**	**On SARS-CoV2**: ‘COVID-19 symptoms’ ‘SARS-CoV2 AND cytokine release syndrome’ ‘SARS-CoV2 entry mechanism’ ‘SARS-CoV2 AND galectins’ ‘SARS-CoV2 S1-NTD’ ‘SARS-CoV2 spike protein’ ‘SARS-CoV2 cytokine storm syndrome’ ‘SARS-CoV2 sialic acids’ ‘SARS-CoV2 fibrosis’ **On β-coronaviruses**: ‘MERS-CoV entry mechanism’ **On Galectin-3**: ‘Galectins’ ‘Galectin-3’ ‘Galectin-3 AND cytokines’ ‘Galectin-3 AND inflammation’ ‘Galectin-3 AND viruses’ ‘Galectin-3 AND viral infection’ ‘Galectin-3 AND fibrosis’ **On Galectin-3 Inhibitors**: ‘Galectin-3 inhibitors’ ‘TD139’ ‘TD139 pulmonary fibrosis’
**Google** **Scholar**	**On SARS-CoV2**: ‘COVID-19,’ ‘COVID-19 symptoms’ ‘SARS-CoV2 fibrosis’ **On Galectin-3:** ‘Galectins’ ‘Galectin-3 cytokines’ ‘Galectin-3 fibrosis’ **On Galectin-3 Inhibitors:** ‘Galectin-3 inhibitors’ ‘TD139’ ‘Gal-3 clinical trials’

### Risk of bias

To minimize the risk of error, all authors involved assessed the cited studies for quality. To discuss important claims in the article, including that SARS-CoV2 binds sialic acids with the S1-NTD, that Gal-3 is upregulated in human immune cells, and Gal-3 inhibitors’ ability to reduce fibrosis, multiple sources were included. Additionally, the use of open-ended searches ensured that an accurate profile of results was obtained on the topics discussed.

## Data availability

No data are associated with this article.
