# Supplementary material for: Immunopathology of galectin-3: an increasingly promising target in COVID-19
Source: F1000Res. 2020 Sep 28;9:1078. Originally published 2020 Sep 1. [Version 2] doi: 10.12688/f1000research.25979.2 (PMC7536583; doi:10.12688/f1000research.25979.2)
Supplement: Peer review history

# Peer review: Reviewer response for version 2

**DOI:** 10.5256/f1000research.29504.r72127

**Published:** 2020-10-05

**Authors:** Talia H Swartz

**Affiliations:** 1 Division of Infectious Diseases, Department of Medicine, Immunology Institute, Icahn School of Medicine at Mount Sinai, New York City, NY, USA

I appreciate the authors' revisions and am happy to approve.

Is the review written in accessible language?

Yes

Are all factual statements correct and adequately supported by citations?

Yes

Are the conclusions drawn appropriate in the context of the current research literature?

Partly

Is the topic of the review discussed comprehensively in the context of the current literature?

Yes

Reviewer Expertise:

NA

I confirm that I have read this submission and believe that I have an appropriate level of expertise to confirm that it is of an acceptable scientific standard.

# Peer review: Reviewer response for version 1

**DOI:** 10.5256/f1000research.28671.r70657

**Published:** 2020-09-16

**Authors:** Thirunavukkarasu Velusamy

**Affiliations:** 1 Department of Biotechnology, Bharathiar University, Coimbatore, Tamil Nadu, India

- The review article is clear, concise, and well structured.
- The article highlights the novel discoveries by linking the pathogenesis of COVID-19 with immunopathologic effects of Gal-3. Dual targeting of Neu5Ac-binding domain of SARS-CoV2 and galectin-3 using galectin-3inhibitors could be a very effective approach in reducing the spread, cytokine storm, and post-infection pulmonary fibrosis.
- The quality of the figures in the article is good and clearly explains the concept discussed.
- The authors are requested to address the following specific comments mentioned below:
  1) The authors have mentioned Gal-3 inhibitors could target Neu5Ac-binding domain, thereby reducing viral entry to host cells. Since ACE2 receptors serve as the main entry mechanism ofSARS-CoV2, To what extent Gal-3 inhibitors alone can provide mitigatory effects?. Also, could ACE2 inhibitors be used as adjuvants along with Gal-3 inhibitors?. What are the possibilities?. These need to be explained.
  2) The authors are advised to cite the article *Garcia-Revilla, J. et al., 2020*in their manuscript. The article discusses similar concepts mentioned in the current review by the authors. Therefore, citing it could provide more support to the authors claim.
- Overall the article is well-written and recommended for publication once the minor corrections have been addressed.

Is the review written in accessible language?

Yes

Are all factual statements correct and adequately supported by citations?

Yes

Are the conclusions drawn appropriate in the context of the current research literature?

Yes

Is the topic of the review discussed comprehensively in the context of the current literature?

Yes

Reviewer Expertise:

NA

I confirm that I have read this submission and believe that I have an appropriate level of expertise to confirm that it is of an acceptable scientific standard.

# Response

**Published:** 2020-09-21

**Authors:** Kiran Velpula

**Affiliations:** University of Illinois at Chicago, USA

Response to Reviewer: Thirunavukkarasu Velusamy

- **The review article is clear, concise, and well structured.**
  - We appreciate the above comment. Thank you.
- **The article highlights the novel discoveries by linking the pathogenesis of COVID-19 with immunopathologic effects of Gal-3. Dual targeting of Neu5Ac-binding domain of SARS-CoV2 and galectin-3 using galectin-3inhibitors could be a very effective approach in reducing the spread, cytokine storm, and post-infection pulmonary fibrosis.**
- **The quality of the figures in the article is good and clearly explains the concept discussed.**
- T **he authors are requested to address the following specific comments mentioned below:**
** 1) The authors have mentioned Gal-3 inhibitors could target Neu5Ac-binding domain, thereby reducing viral entry to host cells. Since ACE2 receptors serve as the main entry mechanism ofSARS-CoV2, To what extent Gal-3 inhibitors alone can provide mitigatory effects?. Also, could ACE2 inhibitors be used as adjuvants along with Gal-3 inhibitors?. What are the possibilities?. These need to be explained**
  - The reviewer brings up a great point. To address this question, we have added a citation that shows the detection of a neutralizing antibody against the NTD of SARS-CoV2 S1 protein. This antibody is effective at completely neutralizing SARS-CoV2 cell entry even in the absence of other antibodies that bind the receptor binding domain or ACE2 receptors. With this in mind, it follows that a drug such as a galectin inhibitor targeting the NTD may be effective as a standalone therapy. The reviewer brings up a good point regarding combination therapy, in that combination of NTD / CTD targeted therapies may be a better long term treatment strategy given the mutagenicity of the virus' spike protein.

 

- **2) The authors are advised to cite the article *Garcia-Revilla, J. et al., 2020 *in their manuscript. The article discusses similar concepts mentioned in the current review by the authors. Therefore, citing it could provide more support to the authors claim.**
  - Thank you for providing this citation. We are certainly glad to see other laboratories participating in this field of research. However, given that this article is also a review article and not primary research, we do not see a role for it to be cited in our current review article, which aims to summarize primary research findings regarding galectin-3 and COVID-19.
- Overall the article is well-written and recommended for publication once the minor corrections have been addressed.

# Peer review: Reviewer response for version 1

**DOI:** 10.5256/f1000research.28671.r70959

**Published:** 2020-09-08

**Authors:** Talia H Swartz

**Affiliations:** 1 Division of Infectious Diseases, Department of Medicine, Immunology Institute, Icahn School of Medicine at Mount Sinai, New York City, NY, USA

The authors here describe the role of Galectin 3 in mediating inflammatory cytokine signaling as a possible source of disease pathogenesis in SARS-CoV-2 infection. The review is well written and describes the relevant literature supporting the role of Gal-3 in COVID-19.

 The following suggestions would improve the strength of the work:

- The authors have published a similar article entitled: A potential role for Galectin-3 inhibitors in the treatment of COVID-19 https://www.ncbi.nlm.nih.gov/pmc/articles/PMC7301894/[ref-1 ]; the title of this current article should clearly reflect how these two works are non-overlapping; the authors describe that this is an update of the prior work.
- The authors write: “Recent discoveries have begun to shed light on its role in viral infections (Wang *et al. *2019) but should go into further detail about what role(s) it plays; there is a literature on HIV (Wang 2014, Okamoto 2019, Fogel 1999).
- The introduction provides very little information about Galectin 3 besides that it is an animal lectin and exerts pleiotropic effects. Some more description should be provided as to the function of this molecule, its tissue expression, and any literature about epigenetics as it pertains to infection and inflammation.
- On page 3, the authors state: “Several studies to date have referred to the “galectin fold” present on the NTD of coronaviruses (Behloul *et al.*, 2020; Li, 2016; Li *et al.*, 2017; Peng *et al.*, 2011; Peng *et al.*, 2012; Tortorici *et al.*, 2019). The structures are so similar, in fact, that it is hypothesized that coronaviruses incorporated a host galectin gene into their genome (and then the NTD) at some point in their evolution (Li, 2015).” The structures of ‘what’ are so similar? NTD to Gal-3? This should be more explicitly defined. This should refer back to Figure 1 in Caniglia PeerJ 2020.
- On page 4, the authors state “Notably, a study of nearly 4,000 patients has found the levels of IL-1, IL-6, and TNF-α to be significantly elevated in the sera of patients suffering from severe COVID-19 as compared to those with mild disease (Wang *et al.*, 2020a).” The authors should additionally cite Del Valle *et al.* Nature Medicine 2020 that noted similar findings in 1500 patients. ^2^
- Figure 1 does not add richly to this work and perhaps could be a panel combined with Figure 2.
- The authors state “Several studies to date have shown the effects of anti-Gal-3 therapy on cytokine release.” These studied should be cited.
- Figure 2 figure legend should address the tissue sites where Gal-3 is produced in macrophages, monocytes, and dendritic cells. Is it lung? Plasma?
- The authors note on p. 5 “A study found that tests for SARS-CoV2 RNA in the serum of infected individuals did not become negative until a median of 24 days post-symptom onset, with some individuals remaining positive even greater than a month from the beginning of symptoms (Gombar *et al.*, 2020). This indicates that for some, COVID-19 infection may run a particularly long course. Findings such as this have led to the question of whether or not anti fibrotic therapy would be beneficial for such patients (George *et al.*, 2020).” The persistence of SARS-CoV-2 RNA should not be equated with replication competent virus; there is significant literature to suggest residual nucleic acid that does not represent infectious virus. This should not be equated with long term infection or increased risk of fibrotic disease and these patients should not be treated with anti-fibrotic therapy for that reason. Severe lung injury from ARDS would be much more plausible an explanation for fibrotic lung disease than persistent SARS-CoV2 RNA.

Is the review written in accessible language?

Yes

Are all factual statements correct and adequately supported by citations?

Yes

Are the conclusions drawn appropriate in the context of the current research literature?

Partly

Is the topic of the review discussed comprehensively in the context of the current literature?

Yes

Reviewer Expertise:

SARS-CoV-2 infection, COVID-19, viral pathogenesis, inflammatory signaling

I confirm that I have read this submission and believe that I have an appropriate level of expertise to confirm that it is of an acceptable scientific standard, however I have significant reservations, as outlined above.

## References

[1] : A potential role for Galectin-3 inhibitors in the treatment of COVID-19. *PeerJ*.2020;8: 10.7717/peerj.9392 e9392 10.7717/peerj.9392 32587806PMC7301894

[2] : An inflammatory cytokine signature predicts COVID-19 severity and survival. *Nat Med*.2020; 10.1038/s41591-020-1051-9 10.1038/s41591-020-1051-9 32839624PMC7869028

# Response

**Published:** 2020-09-08

**Authors:** Kiran Velpula

**Affiliations:** University of Illinois at Chicago, USA

**Response to Reviewer: Talia Swartz, MD, PhD**

The authors here describe the role of Galectin 3 in mediating inflammatory cytokine signaling as a possible source of disease pathogenesis in SARS-CoV-2 infection. The review is well written and describes the relevant literature supporting the role of Gal-3 in COVID-19.

The following suggestions would improve the strength of the work:

- **The authors have published a similar article entitled: A potential role for Galectin-3 inhibitors in the treatment of COVID-19 https://www.ncbi.nlm.nih.gov/pmc/articles/PMC7301894/[ref-1 ]; the title of this current article should clearly reflect how these two works are non-overlapping; the authors describe that this is an update of the prior work.**
  - The reviewer makes a great point. With the title of this work we are trying to convey a distinction in so far as the prior article explicitly argued for the repurposing of a drug toward COVID-19 treatment. In this article, we are discussing the pathologic effects Gal-3 may exert in severe COVID-19, hence the title.
- **The authors write: “Recent discoveries have begun to shed light on its role in viral infections (Wang *et al. *2019) but should go into further detail about what role(s) it plays; there is a literature on HIV (Wang 2014, Okamoto 2019, Fogel 1999).**
  - Thank you for providing the additional references. We have added a couple sentences to more explicitly detail the roles of Gal-3 in viral infection and have incorporated the findings of Okamato et. al into multiple sections of the review.
- **The introduction provides very little information about Galectin 3 besides that it is an animal lectin and exerts pleiotropic effects. Some more description should be provided as to the function of this molecule, its tissue expression, and any literature about epigenetics as it pertains to infection and inflammation.**
  - The reviewer makes an excellent point. We have added additional sources (Diaz Alvarez et. al 2017; Sato et. al 2009) to provide further introduction to the role of galectins in infections and to reference the cell types Gal-3 is most highly expressed in.
- **On page 3, the authors state: “Several studies to date have referred to the “galectin fold” present on the NTD of coronaviruses (Behloul *et al.*, 2020; Li, 2016; Li *et al.*, 2017; Peng *et al.*, 2011; Peng *et al.*, 2012; Tortorici *et al.*, 2019). The structures are so similar, in fact, that it is hypothesized that coronaviruses incorporated a host galectin gene into their genome (and then the NTD) at some point in their evolution (Li, 2015).” The structures of ‘what’ are so similar? NTD to Gal-3? This should be more explicitly defined. This should refer back to Figure 1 in Caniglia PeerJ 2020.**
  - We have revised the sentence to more explicitly define the structural similarities of Gal-3 and S1-NTD of coronavirus spike proteins. We have also added an additional reference of Caniglia et. al.
- **On page 4, the authors state “Notably, a study of nearly 4,000 patients has found the levels of IL-1, IL-6, and TNF-α to be significantly elevated in the sera of patients suffering from severe COVID-19 as compared to those with mild disease (Wang *et al.*, 2020a).” The authors should additionally cite Del Valle *et al.* Nature Medicine 2020 that noted similar findings in 1500 patients. ^2^**
  - Thank you for providing the additional citation. We will certainly add these findings to the manuscript.

 

- **Figure 1 does not add richly to this work and perhaps could be a panel combined with Figure 2.**
  - Thank you for this comment. We believe Figure 1 to be essential as it details the likely role of the galectin-like S1-NTD in COVID-19 infection. To further highlight the importance of this figure and the NTD, we have added a recent publication (Chi et. al, 2020) which shows a neutralizing antibody against the NTD inhibits viral entry. It then follows that if a drug such as a galectin inhibitor is able to also bind this region of the NTD, it may also inhibit cell entry by SARS-CoV2.
- **The authors state “Several studies to date have shown the effects of anti-Gal-3 therapy on cytokine release.” These studied should be cited.**
  - We have added the appropriate sources to support this statement.
- **Figure 2 figure legend should address the tissue sites where Gal-3 is produced in macrophages, monocytes, and dendritic cells. Is it lung? Plasma?**
  - The reviewer makes an excellent point. We have updated the figure legend to show that Gal-3 is produced in circulating immune cells in the plasma.
- **The authors note on p. 5 “A study found that tests for SARS-CoV2 RNA in the serum of infected individuals did not become negative until a median of 24 days post-symptom onset, with some individuals remaining positive even greater than a month from the beginning of symptoms (Gombar *et al.*, 2020). This indicates that for some, COVID-19 infection may run a particularly long course. Findings such as this have led to the question of whether or not anti fibrotic therapy would be beneficial for such patients (George *et al.*, 2020).” The persistence of SARS-CoV-2 RNA should not be equated with replication competent virus; there is significant literature to suggest residual nucleic acid that does not represent infectious virus. This should not be equated with long term infection or increased risk of fibrotic disease and these patients should not be treated with anti-fibrotic therapy for that reason. Severe lung injury from ARDS would be much more plausible an explanation for fibrotic lung disease than persistent SARS-CoV2 RNA.**
  - The reviewer makes an excellent point here. We have added multiple sources to this section that we believe better characterize the chronic inflammatory signature some COVID-19 patients report months after the initial infection. We have also included an additional citation of an excellent commentary on the concern for development of post-infectious IPF in COVID-19 patients.
